# Interface Strength and Fiber Content Influence on Corn Stover Fibers Reinforced Bio-Polyethylene Composites Stiffness

**DOI:** 10.3390/polym13050768

**Published:** 2021-03-01

**Authors:** Quim Tarrés, David Hernández-Díaz, Mònica Ardanuy

**Affiliations:** 1 Departament of Materials Science ande Engineering, Universitat Politècnica de Catalunya (UPC), Colom 1, 08222 Terrassa, Spain; monica.ardanuy@upc.edu; 2 Serra Húnter Programme, Department of Engineering Graphics and Design, Universitat Politècnica de Catalunya, TR5 Campus Terrassa, 08222 Terrassa, Spain; david.hernandez-diaz@upc.edu

**Keywords:** bio-polyethylene, corn stover, Young’s modulus, composites

## Abstract

Stiffness of material is a key parameter that allows the use of material for structural or semi-structural purposes. Besides, lightweight materials are increasingly calling the attention of the industry. Environmental impact is also increasing in its importance. Bio-based materials produced from renewable sources can be good candidates for structural purposes combining lightweight and low environmental impact. Nonetheless, similar mechanical properties of commodities have to be reached with such materials. In this work, composite materials from corn stover fibers as a bio-polyethylene reinforcement were produced and tested. The effect of coupling agents to improve the fiber–matrix interface has been evaluated. It has been found that coupling agent content influenced the stiffness of the materials, increasing the Young’s modulus and the material processability. The best performance was achieved for a 6% of coupling agent, corresponding to 4.61 GPa for 50 *w/w*% of corn stover fibers. Micromechanics showed the impact of the semi-random orientation of the fibers and the lesser impact of its morphology. It was possible to determine a triangular packing of the composites as a hypothesis for future research.

## 1. Introduction

Commonly used composite materials or reinforced polymers, over the last decades, have been constituted by high-strength fibers (glass, aramid, or carbon fiber) embedded in thermoplastic polymer matrices of fossil origin [1,2,3,4]. The composite materials that currently dominate the market in the field of thermoplastics are glass fiber-reinforced polypropylene and polyethylene [5,6]. The use of natural fibers as an alternative reinforcement to synthetic fibers has attracted the attention of many researchers and scientists due to their advantages. The benefits of natural fibers over glass fibers are low cost, low density, nonskin irritation, reduced energy consumption, lower health risk, renewability, recyclability, and biodegradability [7,8]. Some examples of these fibers are spruce, hemp, or henequen, among others [9,10,11]. Although these materials are suitable for a multitude of applications including construction, sports, packaging, and others, the most important applications are in the automotive industry [12,13,14]. This is mainly due to the constant research towards lightweight materials able to fulfill engineering specifications.

Usually, natural fibers have been obtained from wood sources, but the limited availability of natural resources and deforestation presents agricultural residues as a possible alternative for the future [15,16,17]. Agricultural residues are obtained as by-products of the agricultural production process, offering significant advantages such as the low cost of the material and the elimination of the environmental impact of burning such agroforestry waste in the field [18]. However, the cost of collecting, transporting, and processing these residues for their use may be a disadvantage. The use of agricultural residues can be advantageous for the environment and the economy by adding value to a waste product [19,20]. Within agricultural residues, corn residues are one of the most abundant in terms of production [21,22].

Agricultural residues, similarly to wood resources, can be processed and treated by mechanical, thermo-mechanical, semi-chemical, and chemical treatments used in the pulp and paper industry [23,24,25]. These treatments result in the isolation of the lignocellulosic fibers from the original biomass. In this context, the increase in the severity of the treatment leads to a reduction in the yield of the process, as well as a modification in the chemical composition of the resulting fibers [26,27,28]. According to the 12 Principles of Green Chemical Engineering, enunciated by Anastas and Zimmerman [29], the activity of scientists and engineers must converge in the design of new materials, products, processes, and systems that are beneficial to health and the environment. In this sense, it is better to prevent waste than to treat or clean it once it is formed [30]. Therefore, obtaining high performance pulps will help us to generate the minimum under-waste in waste valorization. Recently, bio-based materials produced from renewable resources have been developed based on bioeconomy and low-carbon economy concepts. Renewable raw materials, such as cellulose fiber and polymers from sugar cane, can be used to manufacture value-added products such as environmentally friendly thermal insulation materials [31].

Currently, public environmental concerns, climate change, and limited fossil resources are encouraging governments, companies, and scientists to find environmentally friendly alternatives to petroleum-based resources. Polyolefins, followed by polyesters, comprise the largest fraction of plastics, corresponding to about 45% of the total world plastics consumption [32]. In this sense, bio-based plastics could make an important contribution to reducing the use and dependence on petroleum, as well as the environmental impact. One of the industrially available bioplastics with the greatest potential for immediate application is bio-polyethylene. This polymer, chemically identical to fossil polyethylene, can be produced from sugarcane [33] and combined with natural fibers from agricultural waste to present a great alternative to current materials.

However, the main issue with natural fiber–polymer composites is the poor adhesion between hydrophilic natural fibers and hydrophobic thermoplastic matrices [34,35]. This leads to undesirable properties of the resulting materials. For this reason, it is necessary to incorporate coupling agents, which allow better interfacial adhesion. In the case of polyethylene, it is common to use short-chain polyethylene functionalized with maleic anhydride (MAPE) to improve the compatibility between the fibers and the matrix [36,37].

In general, the tensile properties of composite materials are significantly increased by incorporating fibers, since the intrinsic properties of the fibers (strength and stiffness) are much higher than those of the matrix [38,39,40]. In the case of structural and semi-structural applications, the most relevant property is the stiffness of the resulting material [41]. It is known that the stiffness of the composite material is given by a multitude of factors including the intrinsic properties of the fibers and the matrix, the volume fraction of reinforcement. However, it is usually considered that it is not influenced by the strength of the fiber–matrix interface [42]. On the other hand, some authors have shown that there is a slight influence of the interface on the final stiffness of the composite material [43].

Elastic properties of composite materials can be predicted from the elastic properties of the fibers and matrix [42,44]. However, the experimental evaluation of Young’s modulus of short natural fibers is expensive and difficult. For this reason, these properties are usually predicted by micromechanics models. The most common models for short fiber composites are a modified rule of mixtures [45], the Hirsch model [46], and the Halpin–Tsai and Tsai–Pagano models [47,48]. From the theoretic perspective, fractal models are frequently used for the characterization of properties of fiber-reinforced polymer composite such as tensile properties, elastic properties, etc [49].

In this work, the influence of corn stover fibers obtained by high-performance treatments (thermomechanical pulp) on the stiffness of bio-based polyethylene composite material was studied, using different percentages of coupling agent. This study presents the use of an agricultural residue as a substitute for synthetic fibers with an environmental, technological, and economic advantage. However, the fibers obtained from corn stover have an intrinsic stiffness much lower than that of synthetic fibers. Besides, their high hydrophilicity makes it essential to optimize the fiber–matrix interface to make the resulting material technically competitive. In optimizing the interface and the properties of the composite, the coupling agent content and reinforcement content were in focus. The main objective was to optimize the fiber–matrix interface. There is little literature on the influence of the coupling agent content on the stiffness of the composite with fibers from agricultural residues. For this purpose, BioPEs were reinforced with different contents of corn stover fiber and coupling agent, and the stiffness of the composites was tested.

The results obtained were used for their micromechanics modeling and were compared with the results of fiberglass high-density polyethylene (HDPE) composites, as well as with the results obtained for wood–HDPE composites. Through the study of micromechanics, the intrinsic modulus of corn stover fibers and the average angles of fiber orientation within the material were obtained.

## 2. Materials and Methods

### 2.1. Materials

The composite materials were obtained from a bio-polyethylene (BioPE) matrix, reinforced with corn stover fibers (CSF), and maleic anhydride polyethylene (MAPE) as the coupling agent. The bio-polyethylene (SHA7260) was supplied by Braskem (Sao Paulo, Brazil). According to the supplier, it is an injection-grade polyethylene with a molecular weight of 61.9 g/mol, a density of 0.955 g/cm^3,^ and a melt flow rate (190 °C/2.16 kg) of 20 g/10min. Maleic anhydride polyethylene (Fusabond MB100D) was purchased from Eastman Chemical Products (San Roque, Spain). Corn stover was kindly provided by Fundació Mas Badia (La Tallada d’Empordà, Spain). All chemical reagents used were purchased from Scharlab Spain (Barcelona, Spain) and used as received.

### 2.2. Methods

**Figure 1:**
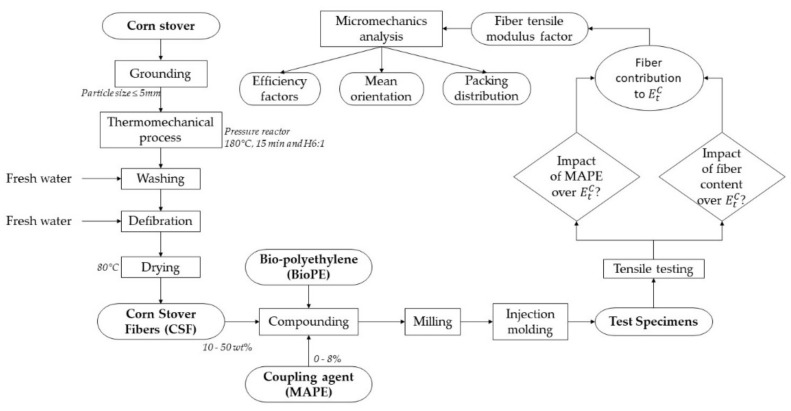
General flow diagram of the present study.

Figure 1 shows the flow diagram of the process of the present study, from fibers extraction and their incorporation as reinforcement of the fully biobased composite materials to their characterization and modeling.

#### 2.2.1. Fiber Isolation from Corn Stover Waste

Initially, the corn stover residues were ground by a blade mill (Agrimsa, Villarrobledo, Spain) to obtain a particle size below 5 mm. The particles obtained were treated by a thermomechanical process in a pressure reactor at 180 °C for 15 min with six parts of water for each part of corn stover particles. After 15 min, the treatment continued by washing the fibrous material and passing it through a Sprout–Waldron defibrator (Andritz, Spain). The fibers collected at the end of the treatment were filtered to a consistency of 10 wt% and then dried in an oven at 80 °C until constant weight.

#### 2.2.2. Composite Compounding

The composite compound was prepared by using a Gelimat kinetic mixer (Dusatec, NJ, USA). Initially, the corn stover fibers (CSF) were introduced into the kinetic mixer maintaining the screw speed at 300 rpm. Once the fiber was incorporated, the bio-polyethylene and maleic anhydride polyethylene were added, also at a screw speed of 300 rpm. Finally, the feed gate was closed, and the screw speed was progressively increased to 3000 rpm. The temperature inside the kinetic mixer increased gradually until it reached 210 °C after 2 min, at which time the material was discharged. The material obtained was ground by a Restech SM 100 blade mill (Restech Iberia, Llanera, Spain). The granulated composite material obtained was stored at 80 °C in the oven to eliminate moisture.

Compounds with different percentages of TMP corn stover fibers, between 10 and 50 wt% and with different percentages of coupling agent (MAPE) between 0 and 10 wt% have been studied.

#### 2.2.3. Composite Injection Molding and Characterization

The elastic properties of the composite materials were determined by testing injection molding standard dog-bone specimens (ASTM D638-14). To prepare the specimens, an Arburg 220M 350-90U injection molding machine (Lossburg, Germany) was used. The temperature profile during injection was kept constant for all composites at 175, 180, 185, 185, and 190 °C, preventing thermal degradation of the fibers. The injection pressure was increased from 300 to 600 bar as the fiber content of the composite increased. The specimens obtained were kept for a minimum of 48 h at 23 °C and 50% relative humidity before evaluation of their properties (ASTM D618).

The specimens were tested on an Intron TM 1122 universal testing machine (Norwood, MA, USA). A load cell of 5 kN and a testing speed of 2 mm/min according to ASTM D790 standard were used. For more accurate values, the test system was equipped with an MFA2 LBG extensometer (Bergamo, Italy). All tests were performed for a total of 10 specimens and the results presented are those obtained from their average and standard deviation.

Analysis of the variance was performed by using R and RComander. The test was made at a 95% confidence level. A multiple contrast of the mean values was made to determine the statistical significance of the differences between the mean values.

## 3. Results

Table 1 presents the Young’s moduli and deformations at break of the composite materials and the effects of fiber and coupling agent percentages over such moduli.

The table shows an increase of Young’s moduli of the composites against increasing percentages of reinforcement. The table also shows a minor variation of Young’s moduli of the composites at the same rate of reinforcement against MAPE contents. Thus, two main factors impacted Young’s moduli of the composites—the percentage of reinforcement and the percentage of coupling agent. Such impacts will be discussed in this paper.

Similarly, the deformation at the break of the composites is highly impacted by the percentage of reinforcements and by the presence and percentage of coupling agents. Besides, while adding a reinforcement has the expected effect on the mechanical properties, the inclusion of a coupling agent and its dosage must be researched to determine the adequate dosages and under what circumstances it is better to use an uncoupled composite over a coupled one.

### 3.1. Impact of the Coupling Agent Content Over the Young’s Modulus

The literature shows two different theories about the impact of the presence of coupling agents on the Young’s modulus of a composite. Some authors minimize such impact and consider it negligible. Nonetheless, these authors does not take into account the standard deviations [50]. Other authors find the impact of the coupling agents low but relevant [43,51].

The analysis of the experimental values obtained in this research revealed that the presence of MAPE had a relevant impact on the Young’s moduli of the composites (Table 1), at least at a 95% rate of confidence from a statistical viewpoint. Table 1 shows how for all the fiber contents, with the exception of 40%, the Young’s moduli of uncoupled and coupled composites with 2% MAPE are not different. Thus, MAPE contents up to 2% have no impact on the stiffness of the composites. However, MAPE contents higher than 2% seem to impact such a property, and a 6% MAPE content seem to deliver Young’s moduli that are different and generally higher than other MAPE dosages. Only in the case of a 10% fiber content, 4% and 6% MAPE contents seem to have similar impacts on the Young’s moduli of the composites. These findings are in agreement with other natural fiber-reinforced HDPE composites [52]. The researchers blame the increase of the moduli on the increasing number of chemical interactions in the interface, mainly ester bonds between the fiber surface and the maleic anhydride [43]. Additionally, the presence of the coupling agent increases the wettability, adsorption-wettability, and molecular entanglement of the fibers by the polymer and the mechanical anchoring [53]. Nonetheless, MAPE contents higher than 6% tend to decrease the Young’s moduli of the composites (Table 1). In the table, it can be observed that, generally, the Young’s moduli of the composites that added 8% of MAPE can be considered different from the other MAPE dosages. This decrease can be due to the presence of excessive MAPE molecules that drive fiber–matrix saturation and MAPE self-bonding phenomena. In this case, MAPE can act as a plasticizer [43]. In any case, it can be concluded that the composites with a 6% MAPE content returned the highest Young’s moduli. Furthermore, adding less than 2% or more than 6% of MAPE is not necessary since in the first case the coupling agent has no impact on the Young’s modulus and in the second case the impact is negative. This is in agreement with prior research [54]. 

Regarding the deformations, it was expected that the presence of a coupling agent increased the potential to deform without breaking the composites. A strong interface increases the load necessary to pull out a fiber, and as a consequence increases its deformation at break. Nonetheless, if the interface is strong enough, and the fibers are supercritical, a fiber break will occur instead of a fiber pullout. In general, composites adding 4% and 6% of MAPE showed the highest deformations at the break, and lesser or higher coupling agent content led to deformation decreases. The literature shows how such deformation variations derived from tensile strength increases and decreases [54].

### 3.2. Impact of the Reinforcement Content Over the Young’s Modulus of the Composites

**Figure 2:**
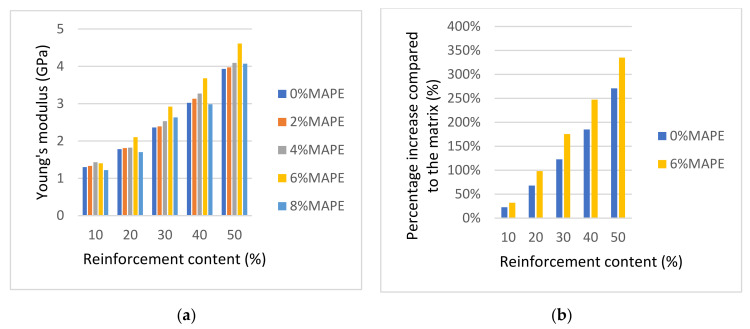
(**a**) Young’s moduli of the composites versus fiber content and (**b**) percentage of Young’s moduli increase in front of the matrix value.

Table 1 and Figure 2a show the increase of the Young’s moduli of the composites against fiber content. In terms of percentage increase of the Young’s moduli, the composites reinforced with 10%, 20%, 30%, 40%, and 50% of reinforcement and 6% of MAPE increased by 32%, 98%, 175%, 247%, and 335% the Young’s modulus of the matrix, respectively. On the other hand, the same uncoupled composites returned 23%, 68%, 123%, 185%, and 271% increases, respectively (Figure 2b).

In the previous section, it was established that a 6% MAPE content delivered the highest Young’s moduli. Figure 2b shows how a coupled composite shows almost the same Young’s modulus as an uncoupled composite with 10% more reinforcement content. This is of importance because the melt flow index of the composites increases noticeably with the fiber content and its ability to be mold injected decreases [54]. Thus, the use of a coupled composite with less fiber content but with analogous stiffness can be advantageous from an industrial point of view.

Regarding the deformation at break, Table 1 shows a noticeable impact of reinforcement contents on such a parameter. The deformations at break decreased noticeably with fiber content. This was expected due to the presence of a more brittle phase and the percentage decrease of the ductile phase. It is worth noting that a coupled composite with a 40% fiber content shows a Young’s modulus only 6% lower than an uncoupled composite with 50% fiber content but has twice the ability to be deformed.

Thus, the use of a coupled agent must be based on its impact on the tensile strength, and the expected deformations under use conditions. In these cases, the percentage of reinforcement has the main role, as it impacts noticeably the stiffness of the composites. Nonetheless, the role of the coupling agent cannot be dismissed as it allows us to reach a similar Young’s modulus and higher ductility with less reinforcement content.

On the other hand, the obtained Young’s moduli are similar to those obtained by other authors that reinforced high-density polyethylene (HDPE) with natural fibers [52]. Thus, BioPE-based composites can subtitutes for HDPE composites reinforced with natural fibers. It must be noted that commodity composites are reinforced with glass fibers. These composites show higher Young’s moduli, due to the intrinsic properties of glass fibers. For example, a Schulman POLYFLAM™ FLP 3714 20% Glass Filled HDPE shows a 2.8 GPa Young’s modulus, and an Aurora Kunststoffe AUROlen^®^ PE-HD GF20 black HDPE, 20% Glass Fiber Reinforced, 2.2 GPa. Thus, it is necessary to add twice the amount of CSF to obtain Young’s moduli similar to GF-reinforced materials. However, this can be seen as an advantage because CSF is less expensive than BioPE and moreover it is a renewable resource.

### 3.3. Contribution of the Fibers to the Young’s Modulus of the Composites

To establish the net contribution of the reinforcements to the Young’s moduli of the composites, the researchers used a modified rule of mixtures (mRoM) defined by the following equation:

(1) $$E_{t}^{C}=\eta_{e}\cdot E_{t}^{F}\cdot V^{F}+\left(1-V^{F}\right)\cdot E_{t}^{M}$$

where $E_{t}^{C}$, $E_{t}^{M}$, and $E_{t}^{F}$ are the Young’s moduli of the composite and the matrix, and the intrinsic Young’s modulus of the fibers, respectively. The Young’s modulus of a semi-aligned short-fiber-reinforced composite is influenced by the morphology of the reinforcements, its interface, and the mean orientation of the fibers. The modulus efficiency factor (${ƞ}_{e}$) equali the contribution of the fibers due to the cited parameters. Finally, *V^F^* is the fiber volume fraction. Equation (1) computes the matrix volume fraction as 1-*V^F^*, and thus dismisses the porosity of the composite.

All the factors of the equation with the exception of $\eta_{e}$ and $E_{t}^{F}$ can be obtained experimentally (Table 2). Thus, the mRoM presents two unknowns and cannot be solved. Nonetheless, $\eta_{e}$·$E_{t}^{F}$ can be defined as the net contribution of the fibers to a semi-aligned short-fiber reinforced composite. This term can be isolated as:

(2) $$\eta_{e}\cdot E_{t}^{F}=\frac{E_{t}^{C}-\left(1-V^{F}\right)\cdot E_{t}^{M}}{V^{F}}$$

**Figure 3:**
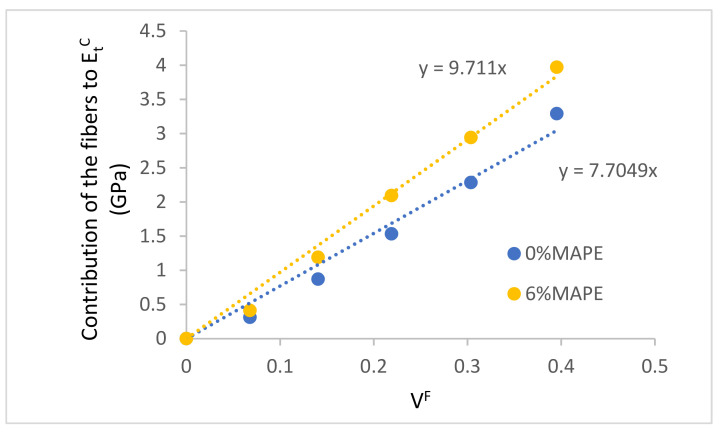
Fiber tensile modulus factor (FTMF) for coupled and uncoupled composites.

Plotting Equation (2) against fiber volume fraction, a linear regression for the obtained points can be obtained. The slope of such a regression line can be related to the stiffening ability of the reinforcement and has been defined as fiber tensile modulus factor (FTMF) [50,52]. Figure 3 shows the FTMF for the uncoupled and 6% coupled composites.

As shown, the contribution of the fibers is different on the coupled with respect to the uncoupled composites. As expected, the stiffening ability of the fibers is enhanced by the presence of MAPE. The figure shows also that the composites at 10% of reinforcement seem to show a lower exploitation of such stiffening abilities. It can be due to its proximity to the minimum critical fiber content [55]. The use of the FTMF makes sense when the values are compared with other fibers as reinforcement of the same or similar matrices. Natural fibers as polyolefin reinforcement for coupled composites return FTMFs in the range from 8.2 to 13.84. The higher values correspond to fibers with high intrinsic properties like jute or hemp [38,39,40]. The lower ones are usually related to recycled fibers or byproduct fibers [56]. CSF is located in the low–middle range of such properties, with potential of reinforcement similar to wood mechanical pulps [57]. Glass fibers as polyolefin return FTMFs around 26.5, showing the stiffening potential of such fibers.

The observed differences between the FTMFs of the coupled and uncoupled composites can be due to the impact of the intrinsic moduli of the fibers or to the modulus efficiency factors. Thus, a micromechanic modeling of the Young’s modulus is proposed.

### 3.4. Micromechanics of the Young’s Moduli of the Composites

Two micromechanics models are proposed to model the contribution of the fiber and matrix phases to the Young’s moduli of the composites. Hirsh’s equation, Tsai and Pagano model and the Halpin and Tsai equations can be used for this purpose [46,48].

Hirsh’s equation is a linear combination of Reuss (parallel) and Voigt (serial) models and can be formalized as:

(3) $$E_{t}^{C}=\beta \cdot \left(E_{t}^{F}\cdot V^{f}+E_{t}^{M}\cdot \left(1-V^{f}\right)\right)+\left(1-\beta \right)\frac{E_{t}^{F}\cdot E_{t}^{M}}{E_{t}^{M}\cdot V^{f}+E_{t}^{F}\cdot \left(1-V^{f}\right)}$$

where *β* equalized the contribution of the serial and parallel models, and a value of 0.4 has been usually used in the literature for semi-aligned short-fiber reinforced composites [50]. Hirsh’s equation only uses experimental values and as soon as two of the moduli and the volume fractions are known, it is possible to obtain the missing module. Then, the equation, together with Table 1 data, can be used to obtain the intrinsic Young’s modulus of the reinforcement.

The Tsai and Pagano model can be formulated as:

(4) $$E_{t}^{C}=\frac{3}{8}E^{11}+\frac{5}{8}E^{22}$$

where *E*^11^ and *E*^22^ are the longitudinal and transversal elastic modulus, respectively. These modules can be calculated using Halpin and Tsai equations:

(5) $$E^{11}=\frac{1+2\cdot \left(l^{F}/d^{F}\right)\cdot \phi_{l}\cdot V^{f}}{1-\phi_{l}\cdot V^{f}}\cdot E_{t}^{M}\;\;\;with\;\;\;\phi_{l}=\frac{\left(E_{t}^{F}/E_{t}^{M}\right)-1}{\left(E_{t}^{F}/E_{t}^{M}\right)+2\cdot \left(l^{F}/d^{F}\right)}$$

(6) $$E^{22}=\frac{1+2\cdot \phi_{t}\cdot V^{f}}{1-\phi_{t}\cdot V^{f}}\cdot E_{t}^{M}\;\;\;with\;\;\;\phi_{t}=\frac{\left(E_{t}^{F}/E_{t}^{M}\right)-1}{\left(E_{t}^{F}/E_{t}^{M}\right)+2}$$

where *l*^F^ and *d*^F^ are referred to as the mean length and diameter of the reinforcements. Both diameter and the length of the reinforcements show high scatter due to their natural source. Such scatter is more noticeable in the case of the diameter than in the case of the lengths, and usually, to obtain sensible results, weighted lengths are used [58]. Its length affects the contribution of the fibers to the Young’s modulus, and such impact is not linear. Besides, usually, longer length fiber population is lower than shorter ones. In this case, the weighted lengths were already used to compute the micromechanics of the tensile strength [54], and the same values will be used here, being 720, 684, 621, 589, and 520 µm for the composites reinforced with 10%, 20%, 30%, 40% and 50% of CSF, respectively.

Table 2 shows the obtained theoretical intrinsic Young’s moduli of the fibers, computed by using Hirsch’s equation and Tsai and Pagano model, and the Halpin and Tsai equations.

The results show two different phenomena. On the one hand, the intrinsic Young’s moduli changed noticeably from coupled to uncoupled composites, being considerably higher for the coupled ones. This can be due to lower exploitation of the stiffening capabilities of the fibers due to weak fiber–matrix interfaces and poor mechanical anchoring between such phases for the uncoupled composites that can develop interface slip even at low deformations. Similar phenomena can be observed when micromechanics are used to compute the intrinsic tensile strength of fibers. Thus, the values obtained for the coupled composites are considered more reliable. For this reason, from here on, only the properties of coupled composites will be considered.

On the other hand, the percentage of fiber influences the intrinsic Young’s modulus of the fiber. It is worth noting that the models used to predict the intrinsic moduli returned similar values.

Table 2 shows how the intrinsic Young’s modulus increases with fiber content. This can be due to the aforementioned possible proximity of the 10% fiber content to minimum critical fiber content. Thus, an increase in the exploitation of the stiffening capabilities of the reinforcement is expected with fiber content. The influence of the proximity of the minimum critical fiber content decreases when the percentage of fiber is increased. Table 2 shows how the intrinsic Young’s modulus of the composites tends to stabilize for the composites with fiber content 30% or higher. Thus, it can be hypothesized that the Young’s moduli of the composites with up to 10% of reinforcement are controlled by the matrix, and the moduli of the composites with higher fiber content are controlled by the fiber. To obtain a mean value for the intrinsic Young’s modulus of the fibers, the values obtained for the composites with 20%, 30%, 40% and 50% have been used and a 18.02 ± 1.1 GPa value has been obtained. This modulus is similar to banana, cotton, abaca, or henequen fibers [59].

Once we know the intrinsic Young’s moduli, it is possible to use the mRoM (Equation (1)) to obtain the modulus efficiency factor. This efficiency factor is the product of an orientation and length efficiency factors (η_e_ = η_o_ · η_l_). The process used to obtain the specimens and its geometry influence η_o_. η_l_ is a consequence of the morphology of the reinforcements [50].

The length efficiency factor can be obtained using Cox and Krenchel’s model [60]:

(7) $$\eta_{l}=1-\frac{\tan\left(\lambda \cdot l^{F}/2\right)}{\left(\lambda \cdot l^{F}/2\right)}\;\;\;with\;\;\lambda =\frac{1}{\left(d^{F}/2\right)}\sqrt{\frac{E_{t}^{M}}{E_{t}^{F}\cdot \left(1-v\right)\cdot \ln\sqrt{\pi /4\cdot V^{f}}}}\;$$

where *λ* is the coefficient of the stress concentration rate at the ends of the fibers and *υ* is the Poisson’s ratio of the matrix.

Finally, the modulus orientation efficiency factor can be obtained as the ratio between the modulus and the length efficiency factors. Table 3 shows the obtained parameters computed from the mean intrinsic moduli obtained with Hirsch’s equation.

The values obtained for the modulus efficiency factor are in the range between 0.47 and 0.49. These values can be considered reasonable as they are in line with the literature [61,62]. However, the values show how only around 50% of the stiffening capabilities of the reinforcement is exploited. The possible reasons for this poor yield in terms of the potential contribution of the fibers to the Young’s modulus of the composite can be attributed to the morphology of the fibers or the morphology of the composite. The fibers showed noticeable mean lengths and a high amount of such fibers were supercritical, ensuring the proper transmission of the loads through the interface and also a regular deformation of the fibers. Length efficiency factors returned high values, always higher than 0.9. Thus, the main cause must be found in the morphology of the composite, specifically in the mean orientation of the reinforcements. Here, the orientation efficiency factors showed values around 0.5, denoting the weight of the orientation of the fibers over the exploitation of their stiffening capabilities. Thus, any procedure able to line up the fibers with the load direction will contribute to composite materials with higher Young’s moduli in such a direction. Nonetheless, such highly oriented composites will show high anisotropy in terms of Young’s modulus.

Measuring the mean orientation of the fibers experimentally is difficult since it is necessary to involve expensive techniques such as X-ray tomography. Another possibility is the use of theoretical models. In this sense, Fukuda and Kawata established a connection between a limit angle (α_o_) and the mean orientation angle of the reinforcements [63]. These authors proposed three possible fiber distributions inside the matrix and the equations to resolve the fiber orientation factor:

Rectangular distribution:

(8) $$\eta_{0}=\frac{\sin\left(\alpha_{0}\right)}{\alpha_{0}}\cdot \left(\frac{3-v}{4}\cdot \frac{\sin\left(\alpha_{0}\right)}{\alpha_{0}}+\frac{1-v}{4}\cdot \frac{\sin\left(3\cdot \alpha_{0}\right)}{3\cdot \alpha_{0}}\right)$$

Sinusoidal distribution:

(9) $$\begin{array}{c} \eta_{0}=\frac{\pi^{2}}{16}\cdot \left(\frac{1}{\pi /2+\alpha_{0}}+\frac{1}{\pi /2-\alpha_{0}}\right)\cdot \cos\left(\alpha_{0}\right)\\ \left[\frac{3-v}{4}\cdot \left(\frac{1}{\pi /2+\alpha_{0}}+\frac{1}{\pi /2-\alpha_{0}}\right)\cdot \cos\left(\alpha_{0}\right)+\frac{1+v}{4}\cdot \left(\frac{1}{\pi /2+3\cdot \alpha_{0}}+\frac{1}{\pi /2-3\cdot \alpha_{0}}\right)\cdot \cos\left(\alpha_{0}\right)\right] \end{array}$$

Triangular distribution:

(10) $$\eta_{0}=4\cdot \frac{1-\cos\left(\alpha_{0}\right)}{\alpha_{0}{}^{2}}\cdot \left(\frac{3-v}{4}\cdot \frac{1-\cos\left(\alpha_{0}\right)}{\alpha_{0}{}^{2}}+\frac{1+v}{4}\cdot \frac{1-\cos\left(3\cdot \alpha_{0}\right)}{9\cdot \alpha_{0}{}^{2}}\right)$$

These equations were used to obtain the limit angles (Table 4). From these angles, Sanomura and Kawamura proposed an orientation parameter (*f*_p_) (Equation (11)) [64] that allows computing the mean orientation angle of the fibers inside the composite (*α*) (Table 4).

(11) $$f_{p}=\frac{\sin\left(2\cdot \alpha_{0}\right)}{2\cdot \alpha_{0}}=2\cdot \cos^{2}\left(\alpha \right)-1$$

As expected, the different fiber packing distributions returned different mean orientation angles. Nevertheless, the main orientation angle can be also obtained from an orientation factor (X_1_) related to the micromechanics of the tensile strength. Such a factor was computed in a prior paper, with values of 0.28 to 0.3 [54]. Having in mind that the orientation factor and the mean orientation angle are related by χ_1_ = cos^4^ (α), these angles will be in the range from 42.4° to 43.3°. The triangular distribution returned mean orientation angles in this range. Thus, to prepare a theoretical model, this distribution seems to be the most adequate. This can help in future studies based on finite model analysis.

## 4. Conclusions

Biobased composites from corn stover fiber-reinforced bio-polyethylene were produced and their Young’s moduli against fiber content and coupling agent percentage were tested. It was found that the percentage of coupling agent influenced the Young’s modulus of the composite. Coupling agent contents in the range from 4 to 6 wt% increased the Young’s modulus. Out of this range, a negative impact on the Young’s modulus was found.

Increases of fiber content provide proportional increases of the Young’s moduli of the composites, despite the use of coupling agents. Nonetheless, the presence of a coupling agent released a similar effect as 10 wt% of reinforcement.

The use of a fiber tensile modulus factor showed that the contribution of CSF to the Young’s modulus of the composite was similar to other lignocellulosic fibers with similar lignin contents.

The modulus efficiency factor informed us that only 50% of the stiffening potential of CSF was exploited. The main reason was attribution to the semi-random orientation of such reinforcement. The mean orientation angle of the fibers obtained by micromechanics analysis was between 42° to 43°. To simulate these composites a triangular packing seems to be a good approach.

The environmental impact of corns stover fiber-based composites is theoretically lower than GF-based materials. Nonetheless, to be completely sure that from an environmental point of view, changing from mineral reinforcements to corn stover fibers makes sense, a more accurate life cycle analysis is needed.

It is necessary to further investigate these composites’ properties such as flexural properties and the effects of water absorption on the mechanical properties of the composites.

## Figures and Tables

**Table 1:** Experimental results of bio-polyethylene composites with different fiber content and different coupling agent dosage. Means followed by the same letter are not a significant difference at the 0.05 probability level.

Fiber Content (wt%)	MAPE Content (%)	$V^{f}$	$E_{t}^{C}$	$\varepsilon_{t}^{C}$	$\rho^{C}$
0	0	0	1.06 ± 0.03	10.59 ± 0.21	0.955
10	0	0.068	1.30 ± 0.02b	6.37 ± 0.23	0.989
	2		1.33 ± 0.03b	6.75 ± 0.06	
	4		1.43 ± 0.02c	6.73 ± 0.14	
	6		1.40 ± 0.02c	6.44 ± 0.16	
	8		1.22 ± 0.02a	6.45 ± 0.23	
20	0	0.141	1.78 ± 0.02f	4.65 ± 0.12	1.026
	2		1.81 ± 0.02f	4.89 ± 0.04	
	4		1.82 ± 0.04f	5.96 ± 0.19	
	6		2.10 ± 0.03g	5.66 ± 0.12	
	8		1.70 ± 0.02h	5.91 ± 0.12	
30	0	0.219	2.36 ± 0.03y	2.58 ± 0.82	1.066
	2		2.39 ± 0.03y	3.02 ± 0.13	
	4		2.53 ± 0.02j	4.65 ± 0.13	
	6		2.92 ± 0.04k	4.90 ± 0.07	
	8		2.63 ± 0.03l	5.13 ± 0.82	
40	0	0.304	3.02 ± 0.03m	1.72 ± 0.03	1.108
	2		3.13 ± 0.02n	2.79 ± 0.22	
	4		3.27 ± 0.04o	4.45 ± 0.28	
	6		3.68 ± 0.03p	4.15 ± 0.08	
	8		2.98 ± 0.03m	4.22 ± 0.03	
50	0	0.395	3.93 ± 0.03q	1.98 ± 0.05	1.155
	2		3.97 ± 0.02q	1.97 ± 0.35	
	4		4.09 ± 0.03r	3.66 ± 0.28	
	6		4.61 ± 0.04s	3.24 ± 0.21	
	8		4.07 ± 0.02r	3.34 ± 0.05	

**Table 2:** Intrinsic Young’s modulus of corn stover fibers (CSF) for coupled an uncoupled composites obtained using micromechanics models.

$V^{f}$	MAPE (%)	$E_{t}^{F}{}_{H}$ (GPa)	$E_{t}^{F}{}_{\mathrm{TP}}$ (GPa)	MAPE (%)	$E_{t}^{F}{}_{H}$ (GPa)	$E_{t}^{F}{}_{\mathrm{TP}}$ (GPa)
0.068	0	8.4	7.2	6	13.2	12.5
0.141		12.2	11.1		17.8	18.3
0.219		14.1	13.1		20.4	21.6
0.304		15.1	14.1		20.5	21.1

**Table 3:** Micromechanics factors for the Young’s modulus of biobased composites.

$V^{f}$	MAPE (%)	$\eta_{e}$	$\eta_{l}$	$\eta_{0}$
0.068	6	0.496	0.925	0.536
0.141		0.474	0.924	0.514
0.219		0.468	0.922	0.508
0.304		0.473	0.929	0.508
0.395		0.476	0.931	0.511

**Table 4:** Limit and mean orientation angles computed from the orientation factor for different packing distributions.

	Rectangular		Triangular		Sinusoidal	
$\eta_{0}$	$\alpha_{0}$	α	$\alpha_{0}$	α	$\alpha_{0}$	α
0.536	55.5	29.1	78.3	40.8	70.8	37.7
0.514	54.4	30.0	81.4	42.0	73.5	38.9
0.508	54.9	30.3	82.3	42.4	74.3	39.2
0.508	54.9	30.3	82.3	42.4	74.3	39.2
0.511	54.7	30.2	81.9	42.2	73.9	39.1

## Data Availability

Not applicable.
